# Integrative Inferences on Pattern Geometries of Grapes Grown under Water Stress and Their Resulting Wines

**DOI:** 10.1371/journal.pone.0160621

**Published:** 2016-08-10

**Authors:** Fushing Hsieh, Chih-Hsin Hsueh, Constantin Heitkamp, Mark Matthews

**Affiliations:** 1 Department of Statistics, University of California at Davis, Davis, CA, United States of America; 2 Department of Viticulture and Enology, University of California at Davis, Davis, CA, United States of America; Wageningen University, NETHERLANDS

## Abstract

Multiple datasets of two consecutive vintages of replicated grape and wines from six different deficit irrigation regimes are characterized and compared. The process consists of four temporal-ordered signature phases: harvest field data, juice composition, wine composition before bottling and bottled wine. A new computing paradigm and an integrative inferential platform are developed for discovering phase-to-phase pattern geometries for such characterization and comparison purposes. Each phase is manifested by a distinct set of features, which are measurable upon phase-specific entities subject to the common set of irrigation regimes. Throughout the four phases, this compilation of data from irrigation regimes with subsamples is termed a space of media-nodes, on which measurements of phase-specific features were recoded. All of these collectively constitute a bipartite network of data, which is then normalized and binary coded. For these serial bipartite networks, we first quantify patterns that characterize individual phases by means of a new computing paradigm called “Data Mechanics”. This computational technique extracts a coupling geometry which captures and reveals interacting dependence among and between media-nodes and feature-nodes in forms of hierarchical block sub-matrices. As one of the principal discoveries, the holistic year-factor persistently surfaces as the most inferential factor in classifying all media-nodes throughout all phases. This could be deemed either surprising in its over-arching dominance or obvious based on popular belief. We formulate and test pattern-based hypotheses that confirm such fundamental patterns. We also attempt to elucidate the driving force underlying the phase-evolution in winemaking via a newly developed partial coupling geometry, which is designed to integrate two coupling geometries. Such partial coupling geometries are confirmed to bear causal and predictive implications. All pattern inferences are performed with respect to a profile of energy distributions sampled from network bootstrapping ensembles conforming to block-structures specified by corresponding hypotheses.

## Introduction

Winemaking can be traced back many thousands of years of human history as a complex process that is intricately intertwined with agriculture and civilizations around the world [1, 2]. The number of mythologies and pseudo-scientific discoveries are manifold and supplemented by a multitude of reports and analyses of specific wines. However, remarkably few systemic and interdisciplinary studies are directed towards the elucidation of definitive global findings. As winemaking continuously evolves alongside human culture, it is perpetually desirable to add new insights based on state-of-the-art technologies. Data-computing and -mining are signature techniques in our current era of Information Technology. In this interdisciplinary study, we attempt to elucidate new perspectives into the winemaking based on new integrative pattern computations and inferences.

These integrative pattern computations and inferences are designed and developed to be applicable on any systemic process, such as winemaking discussed here. Systemic processes are defined as systems comprising large numbers of dynamic subjects observed over extended time periods. The dynamic subject of our choosing are wines, which are specifically defined by the processes of different growing regimes and their standardized processing into wine [3, 4]. Typically, such processes are observed over serial discrete phases consisting of designated temporal periods, for which phase-specific data sets are collected. As such, we study a systemic process involving the simultaneous production of different wines. Our salient interest focuses on discovering and visualizing patterns which characterize constituents of individual phases as well as their respective evolution. Research objectives also focus on identifying possible emerging categorical groups of these dynamic subjects and emerging patterns of their collective features. Our integrative approach is bound to compute pattern geometries and to evaluate whether computed patterns are real (as opposed to mere artifacts.) A computed and confirmed emerging category could offer new insight into winemaking, and the emerging patterns of features might present new knowledge.

From the data structure aspect, one signature phase of a systemic process is characterized by a phase-specific set of features. Hence, individual subject’s data consists of measurements on different sets of features that characterize the individual phases. Such a collection of all involving dynamic subjects is termed a space of media-nodes, while each set of features at each phase is termed a space of feature-nodes. If media-nodes are arranged by rows and feature-nodes by columns of a matrix, then each phase-specific data set can be represented by a matrix of feature measurements. This matrix is usually called a contingency table in statistics literature. However, since the arrangements of media-nodes on row axes and feature-nodes on column axes can be arbitrary, such data sets are invariant with respect to permutations of rows and columns within the contingency table. It is better to precisely refer to such data as a bipartite network. This simple invariance property of a bipartite network turns out to be rather essential in pattern computations. As such, there is a series of phase-specific bipartite networks observed along a target systemic process.

By recognizing the data structure as serial bipartite networks, several conceptual and computational advantages arise. We discuss several such advantages brought forth by bipartite networks not only to differentiate our pattern-based computations and inferences from classic ones based on statistical modeling, but also to highlight why pattern-based approaches are indeed needed. A first conceptual advantage is the ability to disregard the issue of high dimensionality. As a measurable feature at any phase represents one dimension belonging to a media-node, there might be very high dimensionality involving the phase-specific data set. This high dimensionality certainly causes modeling difficulties. One primary source is the lack of suitable knowledge to sustain a coherent modeling platform in order to accommodate multiple dimensional covariate variables in one phase, as well as multiple dimensional response variables in another phase. The limited number of media-nodes relative to the dimensionality of feature-nodes of either covariate or response makes statistical modeling difficult because of missing suitable distributional or manifold constructs. Another source of difficulty is the absence of an effective protocol accommodating unknown structural dependence among involving feature-nodes in modeling. In contrast, there is no need for modeling under the network framework, since our goal is to extract pattern geometry. In order to extract patterns from a bipartite network, a reasonable empirical measure of “similarity” or “distance” among media-nodes is needed. Subject matter knowledge ranks secondary for purposes of mere pattern exploration.

Explicit authenticity of computable pattern formation presents another advantage. The aforementioned unknown structural dependences exist among feature-nodes of each phase, and accordingly among media-nodes as well. This effect is real because all media-nodes are bound together by sharing environmental conditions of the systemic process. Therefore, authentic patterns embedded within a bipartite network are in fact interacting dependence structures among and between media-nodes and feature-nodes. This realization is essential for pattern computations. Furthermore, real-life systemic processes usually contain multiple-scale manifestations. That is, the configuration of such interacting dependences should take a hierarchical format. Such multi-scale structural information appears highly data-dependent, which inhibits a-priori elucidation. Therefore, a data-driven computational approach promises a higher potential for authentic patterns in bipartite networks.

A third advantage lies in the fact the pattern-based information content of a bipartite network is quantifiable and attainable in a non-parametric fashion. It is recently postulated that a bipartite network is better treated as a thermodynamic system in which the minimum energy ground state tentatively embraces the pattern-information content [5]. Therefore, the task of pattern extraction is transformed into an exploration for a discrete combinatorial optimization. Network computations not only realistically resolve this optimization problem, but also represent the pattern-information content in hierarchical block sub-matrices. In this regard, statistical modeling and probability-based inferences become irrelevant.

The potential for computation of causal and predictive patterns adds a fourth advantage. This indeed is the primary goal of this study. Not only do we seek to obtain characteristic information from each individual bipartite network, but also intrinsic understanding on how serial bipartite networks evolve along the temporal axis. With respect to two temporal-ordered phases, a bipartite network derived from the latter phase is termed a network of response, accordingly the one derived from the former phase is termed a network of covariates. Hence one chief goal of this study is to compute what patterns of response are caused by which patterns of covariates. Vice-versa, which patterns of covariates may predict patterns of response? Accordingly, we develop a coherent computational approach to probe into the dynamics underlying systemic processes.

The significance of computing causal and predictive pattern is seen through the following methodological perspectives: For one, computations for causal patterns lay a technical foundation for solving problems that regression-tree or decision-tree leave unsolved. On the other hand, computations for predictive patterns lay a foundation for solving problems that partial least square (PLS) analysis [6] does not resolve. Our underlying premise proposes that integrative pattern computing and inference make effective use of interacting dependence between media-nodes and feature-nodes. The essential nature of multi-scale interacting dependence between media-nodes and feature-nodes elegantly intertwine through pattern-to-pattern inferences, as will be demonstrated below. Although the classic decision-tree gives rise to a hierarchical tree, it fails to reveal the multi-scale structures between media-nodes and dependence structures among covariate features. While the PLS analysis seemingly matches linear dependences of covariate features to response, it completely misses nonlinear geometry structures. These two reflections together illustrate that feature-dimension reduction should be avoided when exploring for global systemic patterns. Because such global patterns are consisting of media-nodes× feature-nodes interacting-relationships, any reduction of feature dimension implies distorting or destructive effects. This seems somewhat counter-intuitive at first glance, when indeed it is not.

This paper is organized as follows. In the Material and Method section, we present brief background information, experimental design and data collecting methods for the serial bipartite networks derived from four phases of winemaking process: harvest grape data, juice composition, wine composition at bottling and bottled wine. All computational and inferential developments, which are primarily focused on the binary version of bipartite networks, are exclusively illustrated through this evolving process of phases. We detail the computing paradigm, Data Mechanics, from theoretical to operational aspects, and the construction of a platform for pattern inference based on energy distribution profiles. In the Results section, we present emerging geometries identified on phase-wise pattern inferences, and results of causal and predictive patterns inferences. All computed pattern geometries on original weighted version of bipartite networks are also presented alongside with their binary ones. They are purely for contrasting and visual confirmation purposes. It is noted that the pattern inferences on weighted bipartite networks rely on key bootstrapping techniques for weighted matrices. Such developments are not mature yet. More research endeavors on this topic are needed.

As a final remark, few tangible causal or predictive patterns have been envisioned or proposed in the literature on serial bipartite networks so far. This may be understood in light of recent technological developments enabling the quantitative techniques for bipartite networks, which were still by-and-large missing over the past two decades. Currently, advances in Information Technology allow researchers to collect data at unprecedentedly small sampling rates. Many real-world events can now legitimately be modeled as evolving systemic processes. As a result, several keystone phases along the process can be identified and studied in a serial manner. Hence our interdisciplinary developments here become not only desirable, but increasingly demanded in science and business alike.

## Materials and Methods

### Data and Systemic process of Winemaking

We begin with a very brief background of three stages of grape berry growth [7].

**A.** “Stage I” berry growth is marked by cell division and differentiation, with the concurrent accumulation of certain metabolites such as tartaric acid or pyrazines, all of which are thought to be feeding-deterrents.
**B.** “Stage II” is observed as no growth, but the induction of various metabolic pathways inside berries, along with the early phase of sugar accumulation.
**C.** Transition from “Stage II” to “Stage III” is called veraison and observed as berry softening as well as color accumulation.
**D.** Stage 3: any subsequent berry growth during ripening is thought to be exclusively caused by the expansion growth of already existing cells. A plethora of metabolites accumulates in the different tissues either by local metabolism or phloem-import. Sugar, malic acid and polyphenolic compounds are regarded as the most important and most abundant solutes.

Therefore, in classic literature in Viticulture, berry growth is termed to follow a double sigmoid pattern: Stage-I and Stage-III represent the two growth and expansions phases, separated by a lag phase(Stage II) [7, 8]. And the transition from Stage-II to Stage-III at the veraison is considered critical. This time point of veraison is the onset of ripening, during which not only sugar accumulation and polyphenol biosynthesis commence, but also anthocyanins, proanthocyanidins, and flavonols are synthesized via the flavonoid pathway. These flavonoids compounds (anthocyanins, proanthocyanidins, and flavonols) are regarded as one of the most important determinants of quality in red grapes and wines. Color and taste of red wines are strongly related to the amount of flavonoids compounds.

Accordingly, it is desirable to regulate the concentration of flavonoid compounds both in grape-growing and winemaking. So far it is known that vine water status could critically affect the accumulation of flavonoids [3, 9]. Thus, regulating vine water status by means of deficit irrigation becomes a tool for increasing flavonoid content and improving quality in red winegrapes [3, 10]. Timing effect of water deficits on winegrape maturation and the accumulation of flavonoids compounds are reported in [11], and continue to be under inter-disciplinary scrutiny.

**Fig 1:**
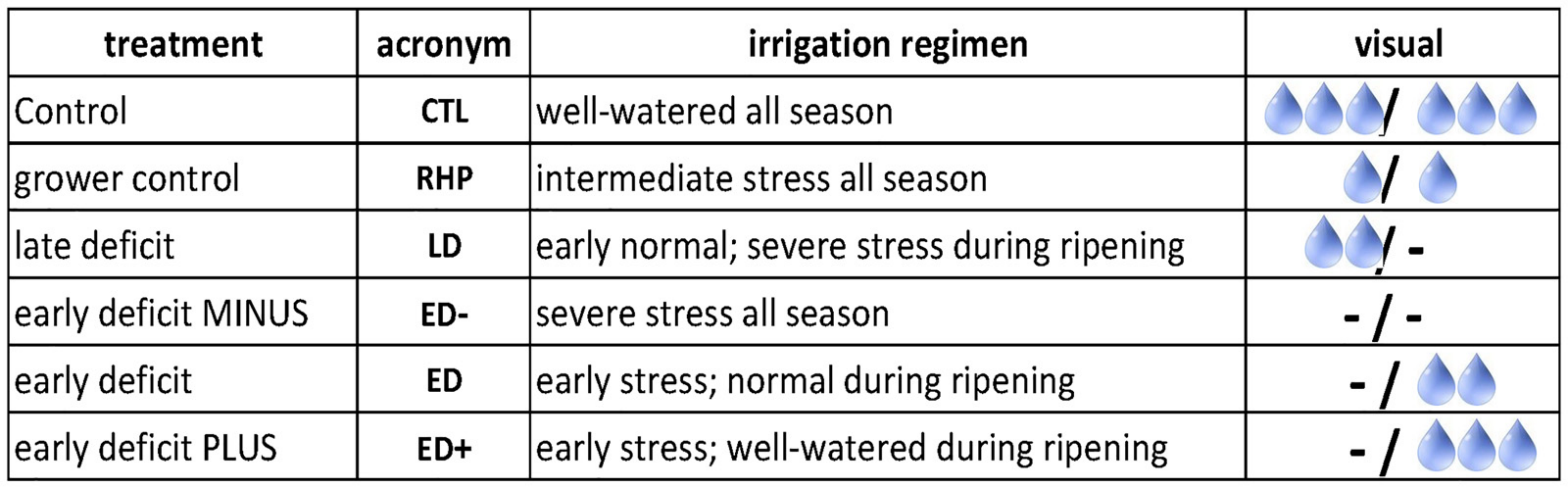
Six water regimes. The slash sign “/” in last column indicates the transition “veraison”, which is typically right at the middle summer.

Our illustrative winemaking system is derived from an experiment revolving around the following six deficit irrigation regimes, as shown in Fig 1: CTL, RHP, ED, ED+, ED- and LD, in their effects on grape composition and winemaking during the 2012 and 2013 vintages. In sequence, the contrasting roles and hypotheses behind each treatment are listed below:

**CTL:** a well-watered control will establish composition at increased yield (leaf-water potential target: -10 bars all season)
**RHP:** the grower control will serve for comparison of practices and targets (leaf-water potential target: -13-14 bars all season)
**LD:** the late deficit will provide context for the early season treatments (leaf-water potential target: -10-11 bars pre-veraison, -14-15 bars post-veraison)
**ED-:** a severe, continued deficit will illustrate grapevine adaptation (leaf-water potential target: -14-15 bars all season)
**ED:** the regular early deficit will juxtapose late season effects (leaf-water potential target: -14-15 bars pre-veraison, -11 bars post-veraison)
**ED+:** will higher yields benefit from ED-induced compositional changes? (leaf-water potential target: -14-15 bars pre-veraison, ¿-10 bars post-veraison)

These treatments were established by the direct plant measurement of stem-water potential (SWP), which were converted into leaf-water potential (LWP) according to [12] for communication with the grower. It is important to emphasize that these experiments used a direct measurement by design to avoid the uncertainties presented by meteorological models such as Evapotranspiration (ET). While ET may be prevalent in traditional, schedule-reliant agriculture, it is also reliant on multiple assumptions. In contrast, direct plant measurements supply a real-time assessment as well as a measure of variability in the field. These measurements lay the foundation for an array of future precision-viticulture applications. While row-crops work reasonably well with ET models, precision viticulture ideally uses direct measurements. It is not possible to convert such direct measurements into equivalents of the theoretical ET model. The use of leaf-water potential measurements for plant water status is recently prevalent among quality (wine)grape-growers.

The experiment site is a Cabernet Sauvignon vineyard located in Esparto, CA. After harvesting grape, the rest of winemaking process was performed in the Pilot Winery of UC Davis. Data sets consist of a series of bipartite networks: from grape composition at harvest through juice analyses, wine composition at bottling and bottled wine data. We analyze the information contents of the four bipartite networks pertaining to these four phases of winemaking. Causal patterns are extracted from the last phase and inferred backward into the second phase, as are predictive patterns from the second phase through the final phase. Though sensory data of trained judges was also collected in the same experiment, the greatly varying nature of human judgments persists as very distinct from the physiological ones analyzed here. As a consequence, sensory data will be analyzed and reported in a separate study.

Below we give a brief description of each bipartite network data.

**Grape composition at harvest** (48 × 14): 14-dimensional measurements of grape chemistry: Acetic, Brix, Citric, Lactic, Malic, pH, Pyrazines, TA, tartaric, A.H-ACY (Anthocyanins by Harbertson-Adams assay), Iland-Acy (Anthocyanins by Iland assay), A.H. IRP (iron-reactive phenolics by Harbertson-Adams assay), A.H.Tannin (tannin by Harbertson-Adams assay), A.H.NTP (non-tannin pigment by Harbertson-Adams assay), at time of harvest, resulting from the respective irrigation treatments with four subsamples. These 14 chemical impact compounds range from ppt-levels (nanograms/kilogram) to the macro-scale of grams/kilogram.
**Juice composition** (36 × 6): the harvested grapes within each treatment were “homogenized” at the winery and split into 3 fermentation batches for each of the six irrigation regimes. The six bio-chemical measurements are: Ammonia, Brix, Malic, NOPA, pH, YAN.
**Wine composition at bottling** (36 × 6): 6 fermentation management features: Acetic, Malic, NOPA, pH, Residual-sugar, Tartaric, were measured for 36 wines resulting from the 36 fermentations of the previous dataset.
**Bottled wine chemistry** (36 × 13): The 36 bottled wines are correspondingly derived from the wines of the previous dataset. The 13 impact compounds: Acetic, Alcohol, A420, A520, FS02, Hue, Intensity, Lactic, Malic, pH, TA, Tartaric, total SO2, were measured.

It is noted that grapes harvested from the same trial blocks were collectively used to make wines. The four field subsamples are connected via water regimes to any specific replicate in the phases of juice, wine at bottling or wine in bottle. In contrast, different replicates of juice are fermented into corresponding replicates of wines. A physical link connects each replicate from juice through bottled wine. That is, the latter three bipartite networks share a common media-node space with 36 dynamic subjects. It is due to such a difference that causal and predictive patterns are computed among phases of juice, wine at bottling and bottled wine. Nonetheless, computed patterns from Harvest field network data still bear causal implications, as will be shown in the Result section.

The original goal of collecting these four bipartite networks of data through four phases of winemaking is to address the issue of how winemaking outcomes are affected by deficit irrigation treatments in the field. Such applied experimental designs are ubiquitous in the pursuit of applied research objectives. However, two surprising perspectives on experimental design and data analytics might alter the way pursuing the practical objective. From the experimental design perspective, though 6 specific irrigation regimes had been implemented on-site, the weather throughout the two seasons presents an uncontrolled factor. Potential interactions between irrigation regimes and weather factors may give rise to complex confounding effects. Even though individual effects of irrigation regimes might be rather difficult to evaluate due to uncontrollable weather factors, a single factor bears overall significance: Vintage.

From data-analysis standpoint, statistical modeling, particularly using parametric models, is expected to meet with prohibitive barriers imposed by high dimensionality. A glimpse of such barriers is seen as follows. There are 14 dimensions of grape biochemicals at harvest as a covariate, and 6 dimensions of juice’s as a response. It is conceivable that unknown dependence patterns exist among the 14-dimensional covariates as well as among the 6-dimensional responses. If it is reasonable to postulate that no sensible effect evaluations can afford completely ignoring dependence structures respectively embedded among both responses and covariate variables, then classic statistics would not be of any good use at all. For instance, most parametric models, such as various regression models, will ignore the dependence among covariates by conditioning arguments, on one hand. One the other hand, hardly any model can accommodate multiple response variables.

Therefore it is logical to turn to nonparametric approaches to be free from imposing unreal structural assumptions and limitations. Then another legitimate question arises regarding sample sizes in such high dimensionality settings: Could only 48 samples constitute a detectable structure in the space of 14-dimensional chemical components? Unfortunately, the almost sure reality is that any 14-dim data cloud of 48 samples indeed hardly affords a manifold with any degree of smoothness. However such smoothness is necessary for building Euclidean geometric manifolds to facilitate pattern recognition approaches.

Thus winemaking as an example system provides a clear reminder that classic statistical methodologies are likely of limited uses in analyzing data from any a complex system. Hence system researchers need to return the origin and fundamental issue: what kind of information is in the data? What kind of pattern-information contents can these four bipartite networks afford us? In this paper we address such a question by use of data-driven computing algorithms. We can reveal patterns embedded within each bipartite networks, and subsequently compute their causal and predictive connections in a global manner. That is, the data-driven computed pattern information allows us to peek into the evolving phases from grape juice to wine.

For expositional and computational simplicity, our primary computing developments and inferencing results are all presented and reported based on binary version of bipartite networks. The binary coding schemes are performed by thresholding at the median, or at an evident valley of a bimodal histogram of biochemical features measured in the four phases. The four sets of histograms superimposed with thresholds are shown in Figs A, B, C and D in S1 File of Supporting Information. Computations and algorithms for extracting patterns on weighted bipartite networks are straight forward extensions of binary developments here. But algorithmic computations for network bootstrapping on weighted bipartite networks are technically much more involved, as would be reported in a separate study.

### Data Mechanics and coupling geometry on binary and weighted bipartite networks

In order to discover the aforementioned interacting dependence structures within a binary bipartite network, we apply the newly developed computing paradigm Data Mechanics. This computational algorithm was originally developed in [5]. One key benefit of working on a binary version first, instead of a weighted one, is its capability for easy visualization. This benefit would allow more thorough computational explorations for identifying and confirming block patterns upon its matrix representation.

#### A physical system perspective

We first quantify the nonparametric information patterns in a binary bipartite network from a physical system perspective. Let a binary bipartite network be represented by an *m* × *n* data matrix as $M_{0}$, with one space of row nodes $X=\{x_{1},..,x_{i},..,x_{m}\}$ and another space of column nodes $Y=\{y_{1},..,y_{j},..,y_{n}\}$. Let $biG(M_{0})$ denote the bipartite network. This network is invariant with respect to the product of row and column permutation groups, denoted as $U_{X}=\left\{\sigma |\sigma =\left(\sigma \left(1\right),\sigma \left(2\right),...,\sigma \left(m\right)\right)\right\}$ and $U_{Y}=\left\{\pi |\pi =\left(\pi \left(1\right),\pi \left(2\right),....,\pi \left(n\right)\right)\right\}$, respectively. A permuted matrix is denoted as $\sigma M_{0}\pi$. From the physical perspective, such a matrix $\sigma M_{0}\pi$ can be taken as a state configuration. That is, the bipartite network $biG(M_{0})$ can be seen as the thermodynamic system defined by the state space of permuted matrices $\left\{\sigma M_{0}\pi |\sigma \in U_{X},\pi \in U_{Y}\right\}$ with total-variation potential. Its Ising ferromagnetic energy level is computed as follows:

$$\begin{array}{c} E\left[\sigma M_{0}\pi \right]=\left(-1\right)\sum_{ij}\sum_{\left(i^{\prime },j^{\prime }\right)\in N\left(i,j\right)}J_{<ij,i^{\prime }j^{\prime }>}\left\{2\left({\left[\sigma M_{0}\pi \right]}_{i^{\prime }j^{\prime }}-1\right)\left(2{\left[\sigma M_{0}\pi \right]}_{ij}-1\right)\right\} \end{array}$$ (1)

where *N*(*i*, *j*) = {(*i*′, *j*′)|*i*′ = *i* ± 1, *j*′ = *j* ± 1} is the set consisting of the four nearest neighbors of the (*i*, *j*) entry on the matrix lattice. Mirroring extensions are required for entries on the lattice edges, and the interaction potential *J*_<*ij*, *i*′*j*′>_ is taken to be constant 1 for simplicity. The Eq (1) defining the energy $E(\sigma M_{0}\pi )$ implies that aggregations of local similarity on the field of *m* × *n* lattices tends to give rise to lower energy levels, while local heterogeneity gives rise to higher energy levels. Therefore, in order to achieve the minimum energy state, this heuristics requires similar row-nodes and column-nodes to be arranged into clusters on both axes. The coupling of one cluster to the row axis and anther to the column axis would form a homogeneous locality in the suitably permuted matrix $\sigma M_{0}\pi$. Such a locality is one way of manifesting interacting dependence structures between spaces X and Y. Ideally we seek for a thermodynamic system state $\sigma M_{0}\pi$ that is basically a patchwork of many such localities.

Such an ideal state configuration achieving the lowest energy level is termed the ground state, or macrostate, of the system $biG(M_{0})$. In statistical mechanics, the macrostate of a system is supposed to reveal the most intrinsic behaviors and patterns of the system. Hence the macrostate establishes the platform to manifest the coherent information content embedded within $biG(M_{0})$. The computational complexity of finding such a macrostate is to solve for the minimizer σ*, π* in the product permutation group $U_{X}\times U_{Y}$:

$$\begin{array}{c} U^{*}\approx \arg\min_{\sigma ,\pi }E\left[\sigma M_{0}\pi \right]. \end{array}$$

In resolving the discrete combinatorial optimization for σ*, π*, any direct search algorithm will encounter computational complexity. Therefore an indirect computing paradigm becomes necessary. This computing paradigm is discussed below.

Before discussing the algorithmic computing, it is worth mentioning that the above systemic concept of a bipartite network fits well in many scientific and real-world settings. Upon this quantification of the minimum energy macrostate, the interacting patterns should be visualized as the nonparametric information content of $biG(M_{0})$. Hence we are confident to reiterate that a bipartite network $biG(M_{0})$ is popularly used to globally approximate a system. Such systems are assumedly shaped by selection forces that render dynamic interactions between row-nodes and column-nodes along an evolving trajectory.

#### Data mechanics: a new computing paradigm

As an indirect search mechanism, the principle of Data Mechanics is to divert the majority of computational complexity into engineering a data-driven surrogate geometric system onto the product permutation group $U_{X}\times U_{Y}$. That is, this surrogate geometry provides this product group with a simple neighborhood system, which would not only allow us to avoid the majority of possible high energy state configurations, but also allow the simple greedy search algorithm to reach the vicinity of the ground state.

This theme can be schematically achieved in a matrix lattice setting as follows: In order to avoid higher energy on the node scale level, we need to group similar rows and similar columns to form core clusters on X and Y, respectively. By grouping similar columns, horizontal segments with homogeneity are created in the correspondingly permuted matrix $M_{0}\pi$. Likewise, grouping similar rows generates vertical segments with homogeneity in $\sigma M_{0}$. Thus, by grouping rows and then columns or vice versa, many small homogeneous blocks are generated on the lattice of $\sigma M_{0}\pi$. Take these small homogeneous blocks to indicate the core cluster scale.

We then re-apply the similarity concept to facilitate the merging of core clusters into conglomerate clusters on X and Y, respectively. Consequently, these joint operations are expected to reduce the total variation-based energy due to emergence of larger blocks on a larger scale. By successively using the similarity idea on various scales, we expect a multiscale block structure to emerge and potentially achieve lower energy levels.

Based on above schematic description, it is obvious that the critical step of applying Data Mechanics on a bipartite network is to build a ultrametric tree on the space of row-nodes and another ultrametric tree on the space of column nodes. These two trees have to be constructed in a highly coupled manner in order to achieve the minimum energy state. The reason behind this is profoundly due to unknown patterns of interacting relationships between the two node-spaces X and Y. These phenomenal, but hidden interactions in fact indicate that the bipartite network data come from a coherent part of the target “complex system”, which is governed by highly structured rules and constraints. These rules and constraints are the target characteristics to be discovered from this network data. Thus, this phenomenal multiscale composition of big or small blocks envisioned in most bipartite networks answers the issues of how and why approximating real a complex system of interest is possible. In summary, Data Mechanics is designed to successfully carry out the discovery of unknown patterns of interacting relationships in a form of mutliscale block patterns embedded within a bipartite network.

The information theory perspective, supplies another reason for the need for two highly coupled ultrametric trees. An ultrametric is a metric satisfying strong triangular inequality. It is known that this property ensures any three points to form either an equilateral or isosceles triangle. An essential implication of this property is the need to treat a core cluster of nodes as a uniform component of point cloud data. Then such a tree could be thought of as a geometric extension of Kolmogorov’s algorithmic statistics with a multiscale structure. Furthermore, by coupling two Ultrametric trees together, we are bound to discover a collection of small or large homogeneous blocks in a matrix representation of a bipartite network. Very importantly, each block signifies a particular interaction relationship between a cluster of row-nodes and a cluster of column-nodes. Accordingly, all locations of blocks jointly marked by the two trees manifest the global structural rules and constraints that we are looking for. This is indeed the essence of Kolmogorov’s two-part coding scheme for transmission of a matrix through a communication channel. Thus, such a data-driven coupling (Ultrametric) geometry would not only constitute the network information content, but also become a very effective tool for visualizing and coding data with high dimensionality.

#### Algorithm of Data Mechanics

Now we propose an iterative algorithm for constructing the pair of ultrametrics $\left(T_{X}^{\left(K\right)},T_{Y}^{\left(K\right)}\right)$ on node-spaces X and Y. At the *k*-th step with *k* = 0, 1, ..*K*, a pair of ultrametrics $\left(T_{X}^{\left(k\right)},T_{Y}^{\left(k\right)}\right)$ is derived based on a pair of updated empirical distance measures $\left(d_{X}^{\left(k\right)},d_{Y}^{\left(k\right)}\right)$. And *K* is determined when the sequence of pair of ultrametrics $\left(T_{X}^{\left(k\right)},T_{Y}^{\left(k\right)}\right)$ converges.

**[Data Mechanics Algorithm]**

**DM1.** Let $d_{X}^{\left(0\right)}$ be the Hamming distance in *n*-dim binary space {0, 1}^*n*^. As $X\subset {\left\{0,1\right\}}^{n}$, compute a *m* × *m* distance matrix and apply the Data-Cloud-Geometry(DCG) algorithm in Fushing et al.(2013) to derive an Ultrametric DCG-tree on X denoted as $T_{X}^{\left(0\right)}$.
**DM2.** Any level of the tree $T_{X}^{\left(0\right)}$ corresponds to a clustering composition of X. Suppose $L_{X}^{\left(1\right)}$ levels (including the bottom level) of the tree are chosen to form a multiscale collection of clusters on X. Denoted this collection as $\left\{C_{X}^{\left(1\right)}\left(l,h\right)|l=1,..,L_{X}^{\left(1\right)}-1;h=1,...,H{\left(l\right)}_{X}\right\}$ with *H*(*l*)_*X*_ number of cluster $C_{X}^{\left(1\right)}\left(l,h\right)$ being on the *l*-th level. The updated distance $d_{Y}^{\left(1\right)}$ from the Euclidean distance $d_{Y}^{\left(0\right)}$ on $Y\subset R^{m}$ is defined as follows:
  $$\begin{array}{c} d_{Y}^{\left(1\right)}\left(y_{i},y_{j}\right)=d_{Y}^{\left(0\right)}+\sum_{i=1}^{L_{Y}^{\left(1\right)}}\sum_{h=1}^{H{\left(l\right)}_{X}}{\left[\mu \left(y_{i}|C_{X}^{\left(1\right)}\left(l,h\right)\right)-\mu \left(y_{j}|C_{X}^{\left(1\right)}\left(l,h\right)\right)\right]}^{2} \end{array}$$
  where $\mu (y_{i}|C_{X}^{\left(1\right)}\left(l,h\right))$ is the sum of components of *y*_*i*_(∈ *R*^*m*^) in $C_{X}^{\left(1\right)}\left(l,h\right)$, so is $\mu (y_{j}|C_{X}^{\left(1\right)}\left(l,h\right)$ for *y*_*j*_.
**DM3.** Take $d_{Y}^{\left(1\right)}$ as the modified Euclidean distance on *R*^*m*^. As $Y\subset R^{m}$, we then compute a *n* × *n* distance matrix, with which an Ultrametric DCG-tree, denoted as $T_{Y}^{\left(1\right)}$, is built on Y.
**DM4.** Take tree $T_{Y}^{\left(1\right)}$ to update the $d_{X}^{\left(0\right)}$ into $d_{X}^{\left(1\right)}$ the same way as in Step-DM2.
**DM5.** Repeat Step-DM1 through Step-DM4 until the sequence of pair of ultrametrics $\left(T_{X}^{\left(k\right)},T_{Y}^{\left(k\right)}\right)$ converges.

The Ultrametric DCG tree computational algorithm used in Step-DM1 is established based on a physical foundation [13, 14], and has been demonstrated via many applications as a more coherent data-driven approach for building clustering hierarchy [15–20]. The Step-DM2 is the heart of the algorithm for Data Mechanics. It prescribes how to operationally couple one tree structural information from a given different node-space onto a target node-space. This one-step coupling turns out very essential and critical in developing computations for causal and predictive patterns. The reason is that it functionally imposes multiscale evaluation criteria into a similarity measure upon the target node-space. We elaborate this in more detail as follows because of its importance in later developments.

For instance, when two major clusters found on a level are included in the collection $\left\{C_{X}^{\left(1\right)}\left(l,h\right)|l=1,..,L_{X}^{\left(1\right)}-1;h=1,...,H{\left(l\right)}_{X}\right\}$, this selection indicates that there exists a significant bifurcation of dependence structure among node-space X. The intuition behind it, according to the DCG-tree on X, the degree of similarity of two nodes is higher when they both are within one cluster than the degree of similarity when they are separated in different clusters. This similarity statement is then translated into node-based dependence. Therefore, this bifurcated structural dependence implies that two distinct characteristics are attaching to these two clusters of member nodes. They have to be summarized separately. Further this intuition implies that two summarizing characters pertaining to these two clusters have to be evaluated and accommodated in the measure of similarity between two feature-nodes. Consequently, the modified Euclidean distance then equips with differentiating capability based on the “two-dimensional summarizing statistics”. For simplicity, we use the simple average as the summarizing statistics.

Thus, with respect to the collection $\left\{C_{X}^{\left(1\right)}\left(l,h\right)|l=1,..,L_{X}^{\left(1\right)}-1;h=1,...,H{\left(l\right)}_{X}\right\}$, the correspondingly modified Euclidean distance is designed to simultaneously perform multiple levels of multiple dimensional comparisons. Such a functional design would become clearer through its application on the winemaking example in the next section. When two Euclidean distances are iteratively modified based on two iteratively coupled trees on X and Y, respectively, the unknown multiscale dependence structures within the bipartite networks $biG(M_{0})$ are extracted and revealed in a form of multiscale block patterns. This version of Data Mechanics algorithm is much more intuitive and computationally simpler than the original version proposed in [5].

Overall, this Data Mechanics Algorithm achieves significant computational advantages in the following manner. After arriving at the pair of ultrametrics $\left(T_{X}^{\left(K\right)},T_{Y}^{\left(K\right)}\right)$, we can directly search for candidate of σ*, π* among the collection of permuted matrices $\sigma M_{0}\pi$ with *σ* being subject to tree $T_{X}^{\left(K\right)}$ and *π* being subject to tree $T_{Y}^{\left(K\right)}$. In fact, this direct search only involves permuting large and small blocks within the matrix lattice. Denote the best row- and column-nodes arrangements as ${\hat{\sigma }}^{*},{\hat{\pi }}^{*}$. Then the permuted matrix ${\hat{\sigma }}^{*}M_{0}{\hat{\pi }}^{*}$ would exhibit multiscale block patterns which are collectively and specifically termed the coupling geometry of the original bipartite network $biG(M_{0})$. Since this coupling geometry reveals mutliscale patterns embedded within a bipartite network, this Data Mechanics is indeed a computational algorithm for “complexity reduction”. Since the formation of our computed block-patterns has taken advantage of inherent interacting dependence structure, Data Mechanics is certainly very distinct from the prevailing concept of dimension reduction in statistics.

### Integrative inferential platform on longitudinal binary networks

As a systemic process goes through its phases in a temporal order, it is natural to wonder how patterns in the earlier phases could possibly cause the computed patterns in the latter phases. Our simple derivation of causal patterns is given as follows. Consider the two phases in temporal order: *P*_*A*_ before *P*_*B*_. Their media-node space is X and feature-node spaces are $Y_{A}$ and $Y_{B}$, respectively. Let $T_{X|Y_{B}}^{\left(K\right)}$ be the Ultrametric tree on X on the coupling geometry of bipartite network of $X\times Y_{B}$ in phase *P*_*B*_.

The simple, but critical idea of computing potential causal patterns is to embed the DCG-tree $T_{X|Y_{B}}^{\left(K\right)}$, which constitutes the coupling geometry on the product node-space $X\times Y_{B}$, onto the product node-space $X\times Y_{A}$ by going through the Step-DM2 of the Data Mechanics algorithm on phase *P*_*A*_. The resultant of DCG-algorithmic computations is called the partial coupling geometry of $X\times Y_{A}|Y_{B}$ in *P*_*A*_ conditioning on phase *P*_*B*_. The reason behind this proposal is that the modification on Euclidean distance imposed by the DCG-tree $T_{X|Y_{B}}^{\left(K\right)}$ would force out causality-bearing feature-patterns to popup on $X\times Y_{A}$. This reasoning can be intuitively seen from the hypothetical case that, if the two final DCG-trees $T_{X|Y_{A}}^{\left(K\right)}$ and $T_{X|Y_{B}}^{\left(K\right)}$ on *P*_*A*_ and *P*_*B*_, respectively, are indeed identical, then the causal relationship between these two phases becomes obvious: the coupling geometry on phase *P*_*B*_ is caused by the coupling geometry found on phase *P*_*A*_. In reality, these two DCG-trees are usually not identical, but have certain degrees of difference. Hence the partial coupling geometry of $X\times Y_{A}|Y_{B}$ differs from the originally computed coupling geometry of $X\times Y_{A}$. Therefore, the question arises: how different are they to confirm or nullify this causal relationship? An answer to this question can be derived from the following energy-based foundation, which is theoretically and experimentally built on bipartite network bootstrapping.

**[Theoretical and experimental foundation for inferential platform:]**

**Theoretical foundation:** Each scale of a computed mutliscale coupling geometry on $X\times Y_{A}$ embraces a structural composition of blocks, and in turns renders a bootstrapping ensemble. The finer scale of coupling geometry contains more structural block information and less randomness. Hence there are more, but smaller blocks in its corresponding composition, which then gives rise to a smaller bootstrapping ensemble. On the opposite, the coarser scale of coupling geometry contains less structural information and a larger amount of randomness. There are less, but larger blocks in its composition, which give rise to a larger bootstrapping ensemble. In summary, a profile of bootstrapping ensembles exists from small to large, pertaining to the computed coupling geometry of $X\times Y_{A}$.
**Experimental foundation:** Consider the 2 × 2 checkerboard-switching as a perturbation process starting from the originally computed coupling geometry of $X\times Y_{A}$. At each time point of the process, a 2 × 2 checkerboard is constructed by randomly selecting two rows and two columns, and satisfying all row and column sums being all equal to 1. Then, its two diagonals are switched. Though keeping the row and column degree sequences unchanged, such a perturbation procedure on a computed coupling geometry would gradually erode the multiscale block-pattern structure. Since a coupling geometry achieves a minimum energy level, this process of eroding can be seen through its energy trajectory starting from its lowest value, then quickly shooting up in value, and finally leveling-off with volatility. This energy leveling-off manifestation indicates that the perturbation process has entered the state in which all perturbed matrices are random matrices in the sense of containing no evident block structural information, but only being subject to the row-degree and column-degree sequences of the observed binary matrix representing the bipartite network.

To make use of the theoretical foundation for inferential purposes, we need to be able to compare two bootstrapping ensembles. Any direct comparison between two such ensembles is in general not practical. But indirect comparisons based on summarizing characteristics of ensembles are available. One summarizing characteristic of an ensemble is its size or cardinality. In theory, the ensemble size can be estimated. However, the knowledge of this estimation is still not yet mature. Another summarizing characteristic is its energy distribution, which is feasible. We know well how to compare two distributions. Therefore, a profile of bootstrapping ensembles pertaining to a coupling geometry on $X\times Y_{A}$ would be manifested by a profile of energy distributions. Such a profile of energy distributions would serve as the landmarks for the coupling geometry. And through the shooting-up pattern of the calculated energy, the Experimental foundation reveals its link to the theoretical foundation. It does so by indicating that most of its bootstrapping ensembles are too small to attract the perturbation process, especially when a coupling geometry exhibits evident multiscale block patterns. That is, the leveling-off states correspond to bootstrapping ensembles of large scales [5].

Thus, an inferential platform based on theoretical and experimental foundations can be outlined as follows: for purposes of confirming or nullifying causal patterns, various energy distributions of several small scales of the partial coupling geometry of $X\times Y_{A}|Y_{B}$ are superimposed onto the profile of landmark energy distributions derived from the computed coupling geometry on $X\times Y_{A}$. If energy distributions of partial coupling geometry are well overlapping with those of small scales in the profile- in other words, they are nested within the very first shooting-up energy range, then we can confirm that patterns of features in $Y_{A}$ can be causally attributed to the formation of DCG tree $T_{X|Y_{B}}^{\left(K\right)}$ with feature patterns in $Y_{B}$. One significant advantage of this inferential platform is that we can calculate the p-values when we investigate different causal patterns by means of setting up simple-vs-simple hypothesis testing problems.

In a similar manner, potential predictive patterns are computed in a reverse order of the causal patterns. In this case, we want to elucidate how patterns in the earlier phases could possibly predict patterns in the latter phases. Again, we consider the two temporal-ordered phases: *P*_*A*_ and *P*_*B*_. Let $T_{X|Y_{A}}^{\left(K\right)}$ be the Ultrametric tree on media-node space X on the coupling geometry of bipartite network of $X\times Y_{A}$ in phase *P*_*A*_. The tree structure $T_{X|Y_{A}}^{\left(K\right)}$ is used as in the Step-DM2 of the Data Mechanics algorithm on the bipartite network of $X\times Y_{B}$ in phase *P*_*B*_. The resultant of DCG-algorithmic computations is called the partial coupling geometry of $X\times Y_{B}|Y_{A}$ in *P*_*B*_ conditioning on phase *P*_*A*_. Once more, the predictive patterns are those confirmed by structural hypotheses testing via bootstrapping ensembles based energy distributions, as discussed previously for causal patterns.

#### Link to discovery

As the coupling geometry ${\hat{\sigma }}^{*}M_{0}{\hat{\pi }}^{*}$ would exhibit multiscale interacting patterns embedded within a bipartite network $biG(M_{0})$. The corresponding tree $T_{X}^{\left(K\right)}$ on the media-node space X could reveal surprising clustering results. Here, the surprising aspect refers to the phenomenon that one clustering configuration on one certain tree level, say *l*_⋆_, unexpectedly turns out matching nearly perfectly with categories of a known factor, say *F*_⋆_. The likely surprising factor *F*_⋆_, which is outside of the feature set Y, is holistic and should be taken as an emerging insight, so it constitues a new discovery. That is, the clustering configuration $\left\{C_{X}^{\left(K\right)}\left(l_{⋆},h\right)|h=1,...,H{\left(l_{⋆}\right)}_{X}\right\}$ on media-node space X marks *H*(*l*_⋆_)_*X*_ categories of “the factor *F*_⋆_”. The surprising knowledge coming out of the identification of *F*_⋆_ is embraced by the categorized collective behaviors, which are characterized by corresponding block patterns of coupling geometry ${\hat{\sigma }}^{*}M_{0}{\hat{\pi }}^{*}$ pertaining to the specific *l*_⋆_ level of tree $T_{X}^{\left(K\right)}$ against all levels of tree $T_{Y}^{\left(K\right)}$ on Y. That is, collective behaviors of recognized factor *F*_⋆_ are quantified as the interaction relational patterns between the *l*_⋆_-level clustering composition of X and multiscale clustering compositions of Y.

It is worth emphasizing again here that this recognition or identification of “the factor *F*_⋆_” is not a result of variable selection in statistics literature, because any level clustering configuration in the collection of $\left\{C_{X}^{\left(K\right)}\left(l_{⋆},h\right)|h=1,...,H{\left(l_{⋆}\right)}_{X}\right\}$ is a computational result of holistic nature due to “row-cluster vs column-cluster” interactions. That is to say, the *l*_⋆_ level clustering configuration is a very complex function of feature-nodes of Y. Can it be computationally recognized and identified? Our conclusion is positive.

We demonstrated this capability via causal and predictive pattern computations through the real data illustration in the result section below. The utilized foundation is that the multiscale characteristic patterns of collective behavior would be specifically quantified and confirmed via network bootstrapping techniques. A series of hypothesis with finer vs coarser structural patterns are formulated intuitively and tested by comparing the energy distributions derived from bootstrapping ensembles corresponding to null and alternative hypotheses.

## Results

### Results of phase-wise pattern inferences

In this section, Data Mechanics is applied on each phase of the winemaking. Based on the series of coupling geometries, we first discover two somehow surprising factors that persistently appear on all four computed coupling geometries throughout all phases: one is on the axis of water regimes, the other one is on the axis of biochemical features. These two factors indeed collectively constitute a backbone of each computed coupling geometry. We then make use of network bootstrapping to build energy distribution profiles to confirm which scales of geometric structures are valid. At the end, from causal and predictive perspectives, we build the partial coupling geometries to link between phases. The validation of a phase-to-phase linkage is also performed based on energy distributions.

#### Four pairs of coupling geometries on binary bipartite networks

**Fig 2:**
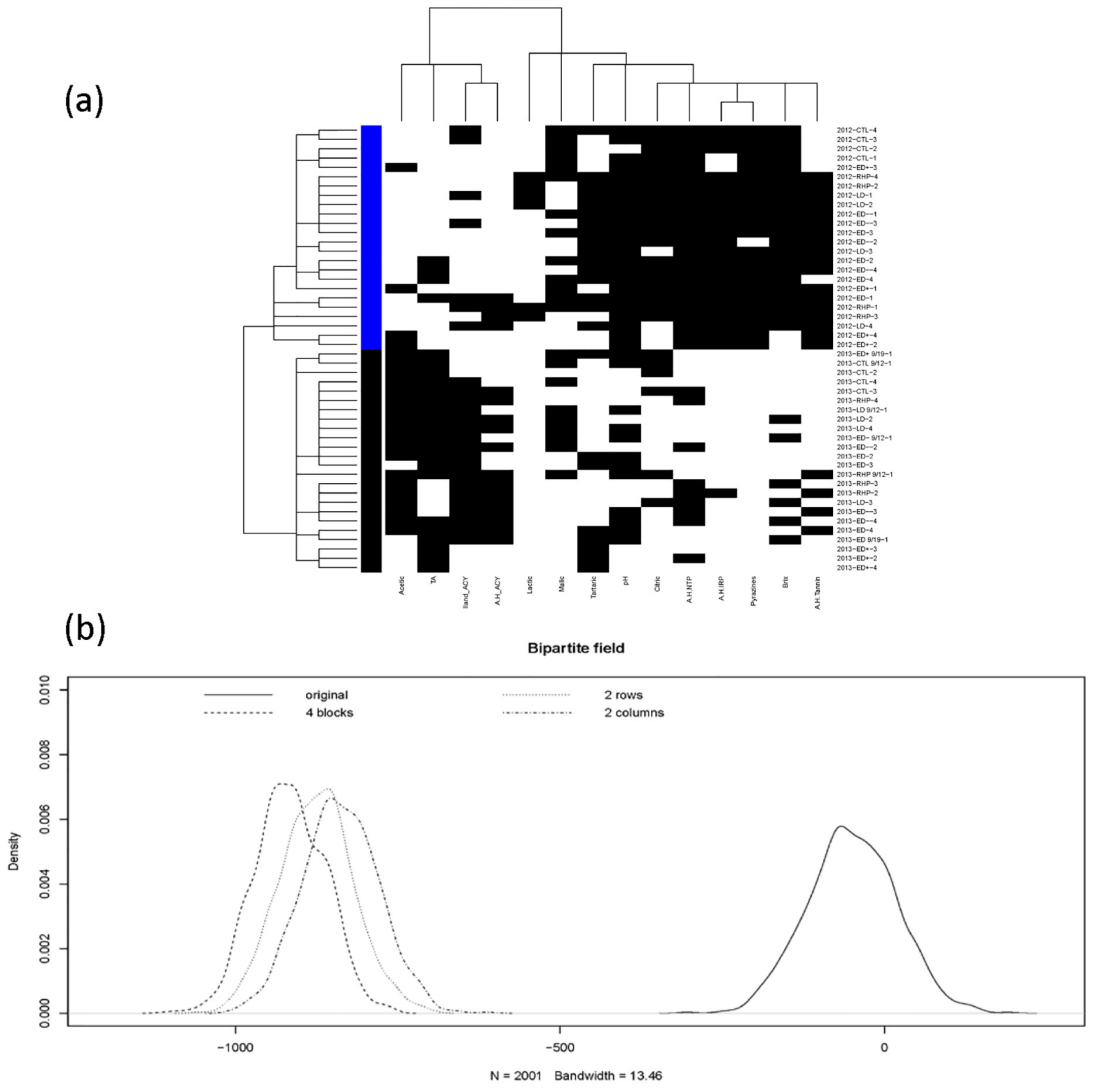
At harvest field phase. (a) Coupling geometries of binary bipartite network, and (b) its profile of four energy distributions upon four structural scales.

In the harvest field phase, the emergent pattern from the coupling geometry of grape’s bio-chemicals, as shown in panel (a) of Fig 2, is surprising. The vintage factor is seen as a collective factor that more effectively binds together various irrigation regimes than the categories of irrigation regimes themselves. This is somehow surprising because the Year factor is holistic, and slightly mysterious. The mystery is in part due to the fact that the 14 attributes are grape’s bio-chemical measurements, which are far from meteorological measurements.

Next, an inferential procedure is proposed to quantitatively check whether various visible computed patterns are real or not. Let the structural pattern of having four blocks be denoted as hypothesis *H*_2 × 2_ to indicate the interacting relationships of binary vintage-factor vs. two-clusters of 14 features. While hypothesis *H*_2*R*_ denotes the marginal structural pattern pertaining to vintage-factor without separating the 14 chemical attributes into two clusters. Similarly hypothesis *H*_2*C*_ denotes the marginal structural pattern pertaining to two-clusters of the 14 features without vintage-factor. The last one is the hypothesis *H*_0_ for without having structural patterns in the bipartite network.

To test among the four hypotheses {*H*_0_, *H*_2*C*_, *H*_2*R*_, *H*_2 × 2_}, we correspondingly generate four bootstrapping ensembles according to their structural information in coupling geometries based on binary bipartite networks. The bootstrapping principle for a binary bipartite network is given in [5]: a bootstrapped network is a pitch-up of all simulated blocks that constitute and bear the structural information. To simulate a binary block subject to row and column sums, the algorithm proposed in [21] and its modified version in [5] is used.

The final step of our proposed inferential procedure is to derive an energy distribution from each bootstrapping ensemble, as shown in the panel (b) Fig 2. Each energy distribution is taken as the corresponding distribution under the hypothesis. Hence for testing among four hypotheses, a set-up of simple vs simple hypothesis testing problem is employed. The *p*−values and type-II errors are calculated by simply evaluating the overlapping areas on either sides of the crossing point of the two corresponding pair of energy distributions. It is seen that energy distributions pertaining to the 4-block and two 2-block patterns are close to each other, while the one pertaining to without structural pattern is very far away. This phenomenon is primarily due to various constraints on block-wise row and column sums. This observation means that the p-values are nearly zeros, so are the type-II errors for *H*_0_, against any one of {*H*_2*C*_, *H*_2*R*_, *H*_2 × 2_}.

In the Juice phase, the coupling geometries based on the binary bipartite network reveals clear 4-block pattern. The vintage factor is even more strikingly evident through the coupling geometry. The 2012 juices from different irrigation regimes are completely separated from those of 2013. It is surprising that this manifestation is retained in juice data. This is certainly a somehow mysterious phenomenon because vintage is not easily characterized by any known function of the 6 chemical features.

**Fig 3:**
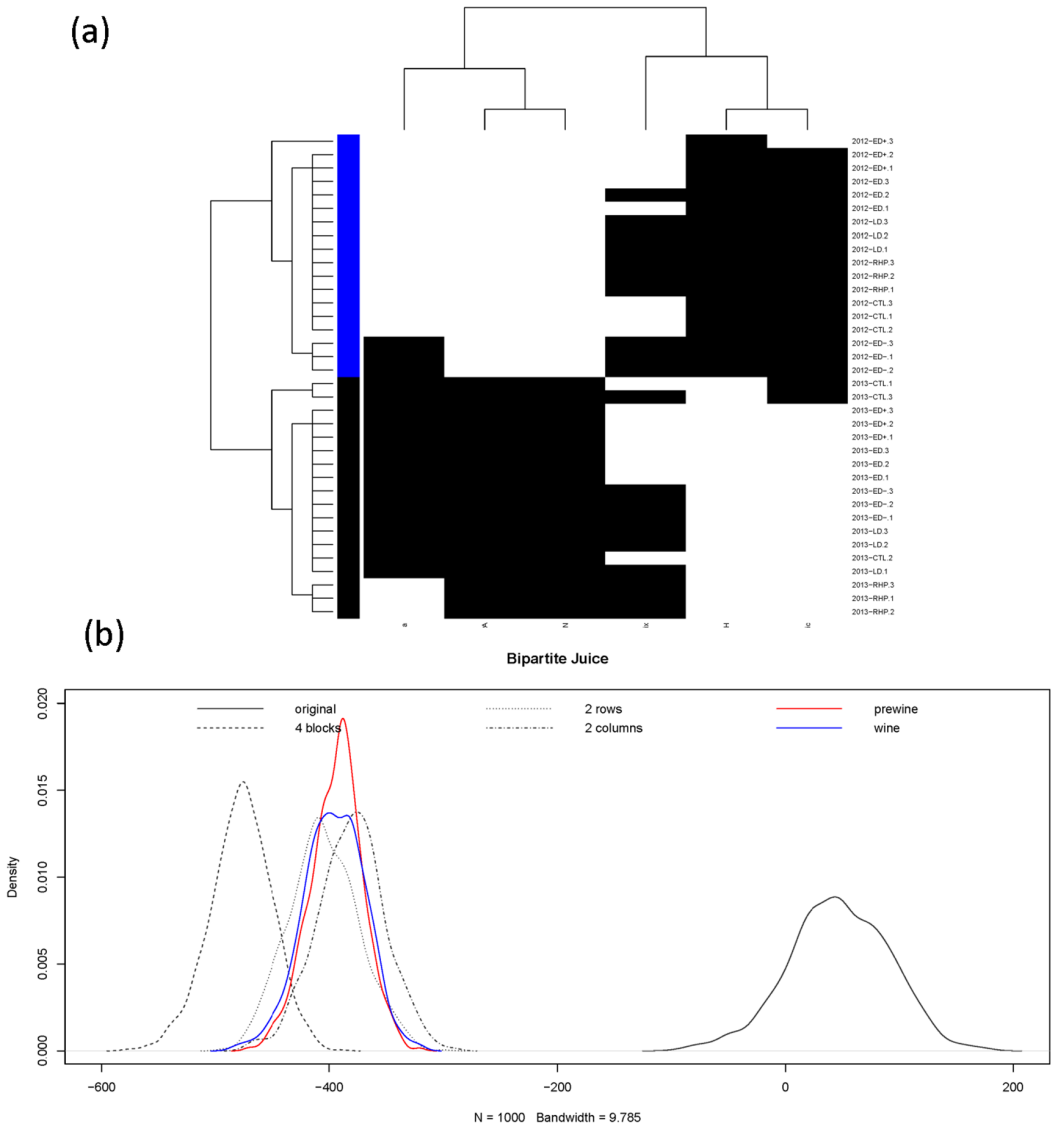
At Juice phase. (a) Coupling geometries of binary bipartite network, and (b) its profile of four energy distributions upon four structural scales together with two energy distributions of two partial coupling geometries constrained by wine-at-bottling (in red) and bottled-wine (in blue).

One intuitive reason behind this phenomenon is that collective interactions between irrigating regimes and the 6 features of 2012 juices were drastically different from the interactions in 2013. This difference is clearly expressed through the evident four block patterns in the heatmap, shown in the panel (a) of Fig 3. This explanation seems very pertinent. If we recognize vintage 2012 and 2013 as two emergent categories, then their collective behaviors are characterized by the block patterns of the coupling geometry.

For quantitatively testing computed patterns on the juice phase, we again consider the four hypotheses {*H*_0_, *H*_2*C*_, *H*_2*R*_, *H*_2 × 2_}. Four energy distributions from four bootstrapping ensembles are derived, as shown in the panel (b) of Fig 3. Again {*H*_2*C*_, *H*_2*R*_, *H*_2 × 2_} are significantly against *H*_0_. So the computed patterns are realistic. Also it is noted that hypothesis *H*_2 × 2_ is apart from *H*_2*C*_ and even farther away from *H*_2*R*_. It implies that the 4-block pattern is significantly different from the two 2-block patterns, while vintage-factor is more important than the two clusters of features.

**Fig 4:**
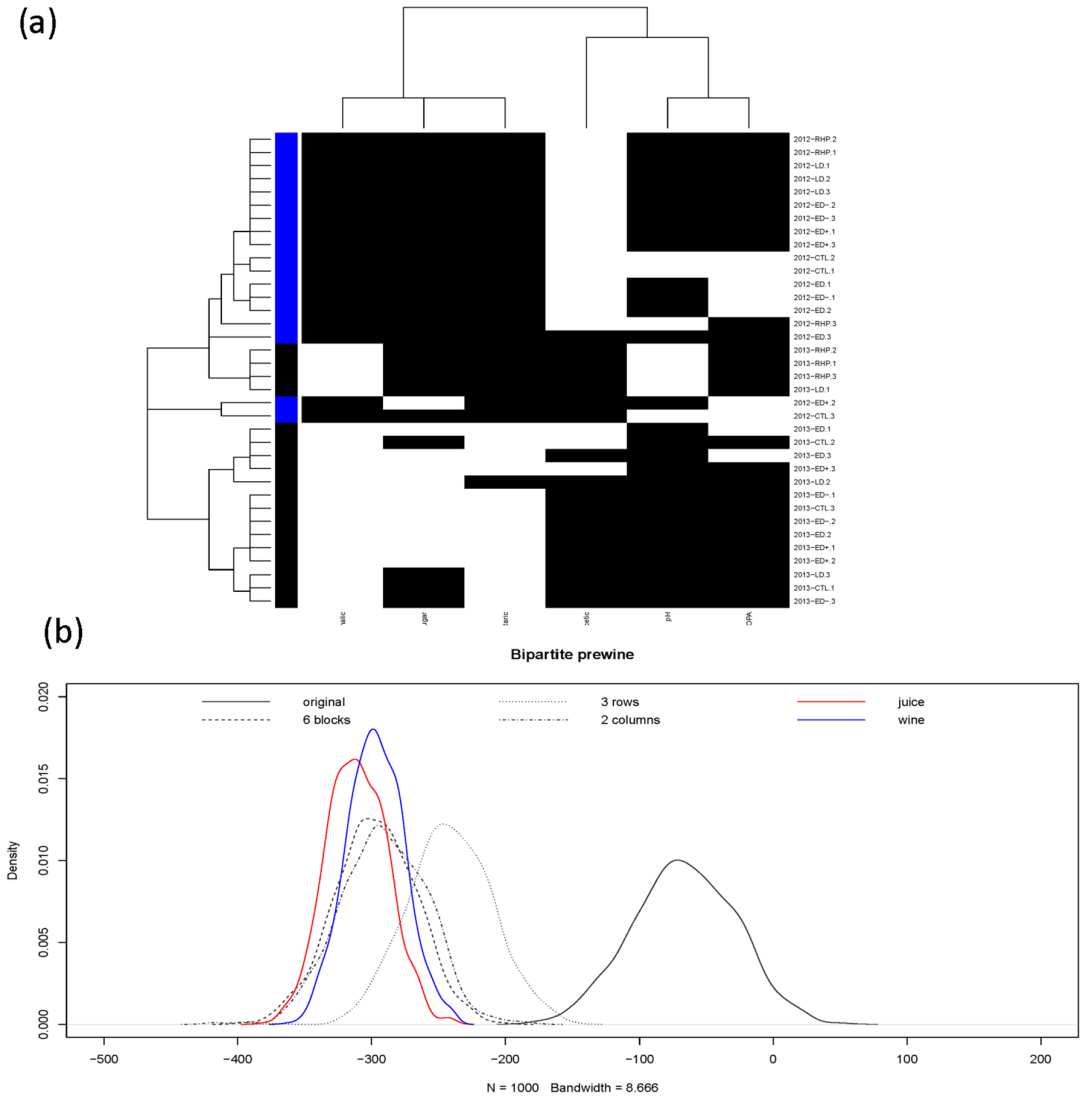
At Wine-at-bottling phase. (a) Coupling geometries of binary bipartite network, and (b) its profile of four energy distributions upon four structural scales together with two energy distributions of two partial coupling geometries constrained by Juice (in red) and bottled-wine (in blue).

Throughout winemaking, the specific features in juice and wine-at-bottling may be termed “housekeeping” measurements since they guide fermentation management. For instance, as a result, YAN was adjusted by addition of nutrients, and pH was lowered by addition of tartaric acid. Both of these adjustments were necessary to guarantee “clean”, reproducible fermentations. At the wine-at-bottling phase, as shown in panel (a) Fig 4, the coupling geometry does not bring out as evident separation between vintages as in the juice phase, but the basic separation remains visible. Similarly, one feature cluster of {*malic*, *residual*−*sugar*, *tartaric*} against the other cluster {*acetic*, *ph*, *nopa*} seems rather clear. For quantitatively testing computed patterns on this phase, we consider the four hypotheses {*H*_0_, *H*_3*C*_, *H*_2*R*_, *H*_3 × 2_}, that is, a small cluster consisting of two 2012 irrigation regimes is added. Four energy distributions from four bootstrapping ensembles are derived, as shown in the panel (b) Fig 4. Again {*H*_3*C*_, *H*_2*R*_, *H*_2 × 2_} are significantly against *H*_0_. Here we note that hypothesis *H*_3 × 2_ and *H*_2*C*_ are rather close, while being away from *H*_2*R*_. Hence the 3-block pattern is significantly evident.

**Fig 5:**
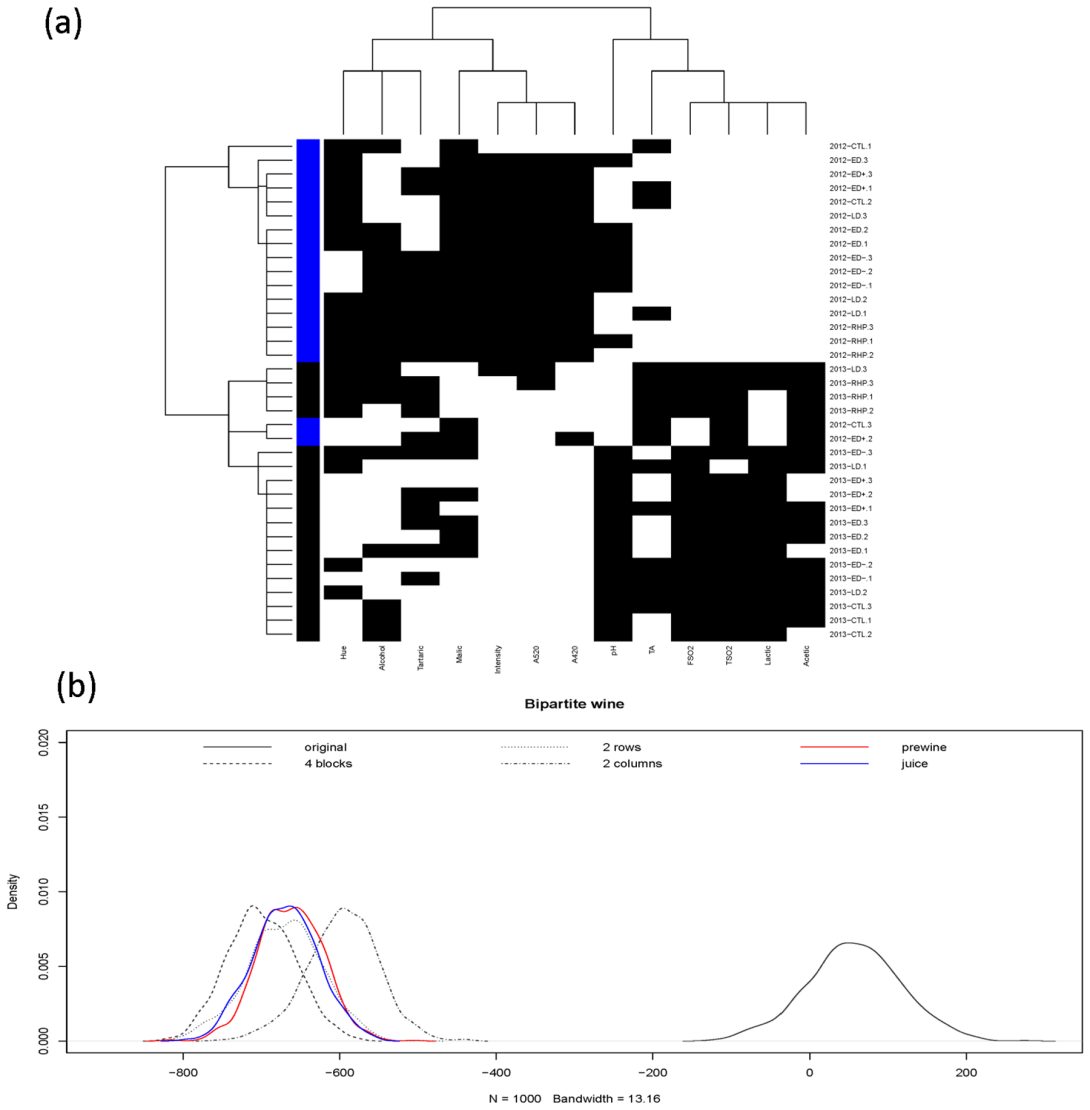
At Bottled-Wine phase. (a) Coupling geometries of binary bipartite network, and (b) its profile of four energy distributions upon four structural scales together with two energy distributions of partial coupling geometries constrained by wine-at-bottling (in red) and juice (in blue).

In the phase of bottled-wine, as shown in panel (a) of Fig 5, the coupling geometry brings out visibly interacting patterns between the vintage-factor and a feature-factor. Based on the four energy distributions derived from its coupling geometry, {*H*_2*C*_, *H*_2*R*_, *H*_2 × 2_} are seen being significantly against *H*_0_, as shown in the panel (b) of Fig 5. But the the separation between *H*_2 × 2_ and *H*_2*R*_ is somehow clear, but not between *H*_2 × 2_ and *H*_2*C*_, nor between *H*_2*C*_ and *H*_2*R*_. Hence, we still retain that vintage-factor is more important than the feature-factor.

The consistent large scale block structure, commonly shown in the above figures, is striking along the four winemaking phases. Such a pattern can be thought of as summarizing statistics that successfully bring out key characteristic differences among one collection of wines and various collections of features by revealing their interacting patterns. Such nearly uniform manifestations strongly indicate that the effects of water irrigating regimes are completely overwhelmed by the holistic interaction effects. In sharp contrast, there are no evident and persistent fine scale patterns throughout the entire winemaking system. Replicates of water irrigating regimes on the rows all heatmaps disperse over the vintage-specific tree branches with different degrees and distinct formations. Such results echo previous studies on effects of irrigation. They have been nicely summarized in part of the abstract in [22]:

“Berry mass and anthocyanin and tannin contents were affected little and inconsistently by irrigation and crop load adjustment and varied mostly among years, indicating a dominant influence of seasonal climate on berry development and composition.”

Though our computational results confirm negative results in [22] on the fine scale aspect, viticulturists and winemakers can utilize the computed system patterns and biochemical factors to redesign and refine their field practices for large scale effects. Such positive and negative aspects of results are potentially essential to system scientists beyond viticulturists or winemakers because of the necessity of recognizing the complexity of conducting experiments in all open or semi-open systems. The expectation of overwhelming confounding effects on experimental regimes is not only real, but also necessary. This is one of many reasons why system sciences are challenging and interesting.

### Results of Causal and predictive pattern inferences

Next we proceeded to compute a series of partial coupling geometries for causal patterns on bipartite networks of covariates, and then a series of partial coupling geometries for predictive patterns on bipartite networks of responses. These two series of partial coupling geometries constitute serial causal and predictive patterns of the winemaking system. Again, energy distributions based on network bootstrapping ensembles were derived to confirm such causal and predictive patterns. Overall, this platform built upon coupling geometry, network bootstrapping and energy distribution is a brand-new inferential paradigm for longitudinal bipartite networks. Their potential applicabilities are expected in diverse scientific fields.

#### Potential causal patterns

**Fig 6:**
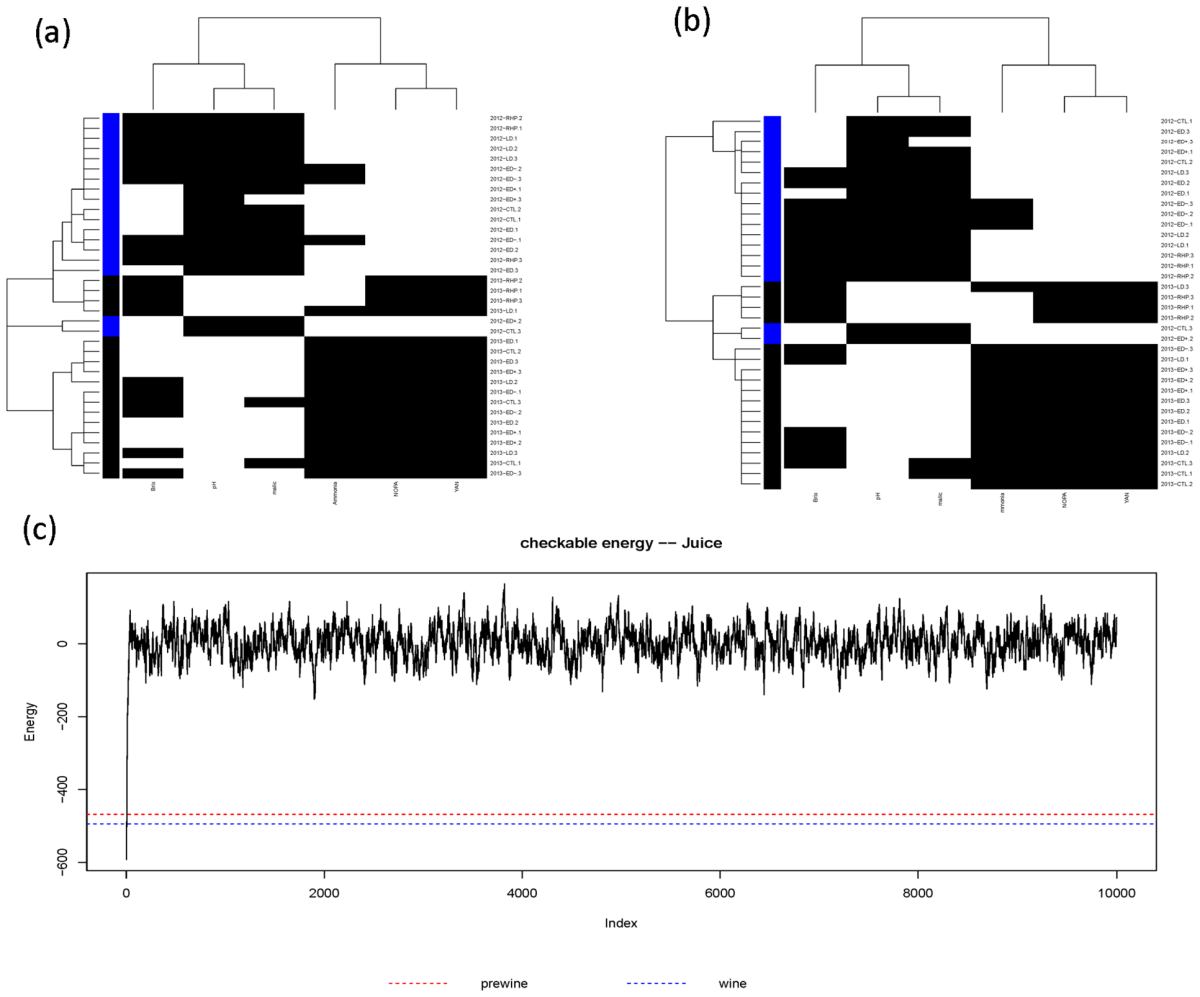
Partial coupling geometries on Juice data matrix constrained by the ultrametric trees of (a) wine-at-bottling and (b) bottled-wine. (c) The energy trajectory of the 2 × 2 checkerboard switching perturbation process starting from Juice’s coupling geometry. Marked lines are Juice’s (minimum) energy = -592, Juice by wine at bottling = -468, Juice by bottled wine = -494.

The partial coupling geometries for causal patterns of wine-at-bottling and bottled-wine (as phases *P*_*B*_) with respect to Juice (as phase *P*_*A*_) are computed and shown in panels (a) and (b) of Fig 6. These two partial coupling geometries still reveal clear block patterns, which are rather similar with the coupling geometry of Juice in Fig 3. One might intuitively assume that such block patterns in Juice are causal patterns for patterns observed in corresponding coupling geometries of the two wine phases. To quantify this statement, energy distributions of the 4-block pattern in partial coupling geometry are derived and compared with the four original energy distributions under hypotheses {*H*_0_, *H*_2*C*_, *H*_2*R*_, *H*_2 × 2_} in Fig 3(the red for wine at bottling, and blue for bottled wine). Both energy distributions are rather close to the energy distribution under *H*_2*R*_. This empirical fact indicates that the vintage-factor of juice is a significant causal factor for the coupling geometries of wine.

A further confirmation via integrative pattern inference is demonstrated in panel (c) of Fig 6. The energy trajectory of the 2 × 2 checkerboard switching perturbation process shows not only the pattern of very steep shotting-up from the minimum energy and then leveling-off, but also the two energies of the two computed partial coupling geometries being far below the leveling-off value. These empirical facts together indicate that Juice’s Year-factor is a significant causal factor for the coupling geometries of wine-at-bottling and bottled-wine.

**Fig 7:**
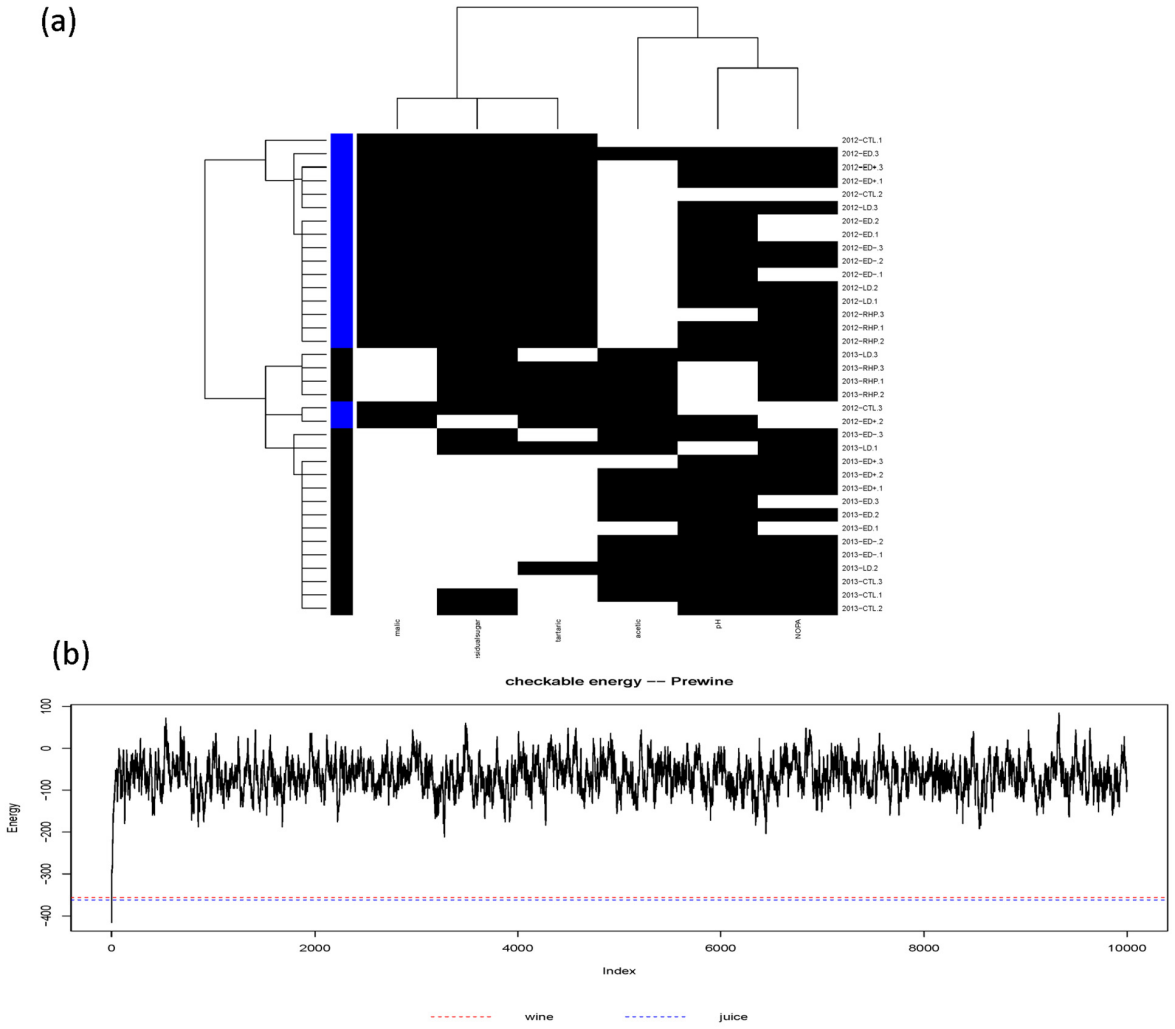
Partial coupling geometries on wine-at-bottling data matrix constrained by ultrametric tree of (a)bottled-wine. (b) The energy trajectory of the 2 × 2 checkerboard switching perturbation process starting from Wine-at-bottling’s coupling geometry. Marked lines are: the minimum wine-at-bottling itself = -416, wine-at-bottling by bottled-wine = -356, Wine-at-bottling by juice = -362.

The partial coupling geometry for causal patterns of bottled-wine with respect to wine-at-bottling shows similar clear block patterns as seen in panel (a) of Fig 7. Its corresponding energy distribution, shown in Fig 4(in blue), is seemingly completely overlapping with that of *H*_3 × 2_. And the calculated energy level is marked near the setting-off level of the energy trajectory of the 2 × 2 checkerboard switching perturbation process, as given in panel (b) of Fig 7. Such pattern linkages on bottled-wine caused by juice and by Wine-at-bottling are somehow expected within winemaking. Hence we demonstrate that the partial coupling geometry and the energy based pattern inference are reasonable and realistic.

#### Potential predictive patterns

**Fig 8:**
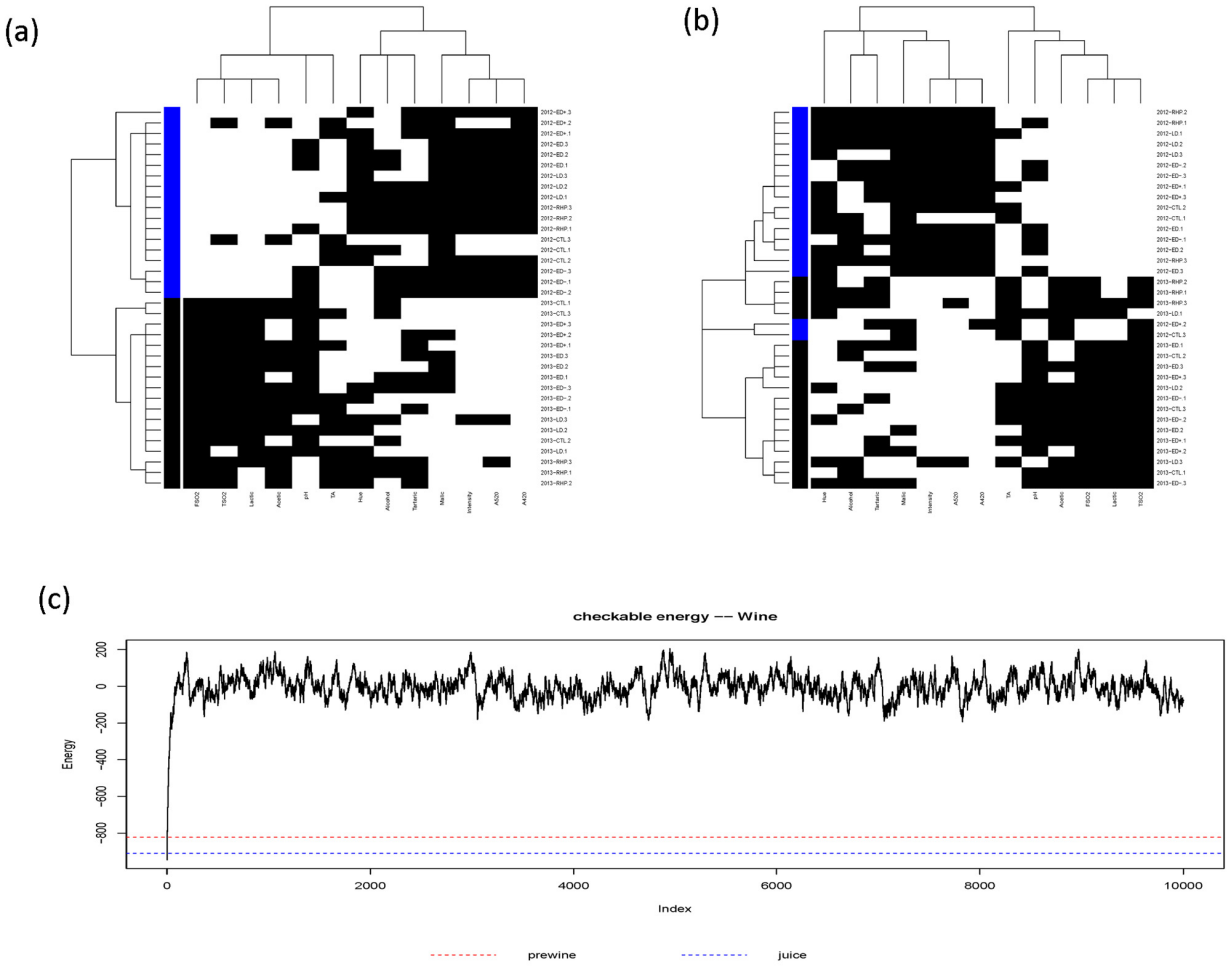
Partial coupling geometries on bottled-wine data matrix constrained by the ultrametric tree of (a) Juice and (b) Wine-at-bottling. The energy trajectory of the 2 × 2 checkerboard switching perturbation process starting from Bottled-wine’s coupling geometry. Marked lines are:(minimum)bottled-wine itself = -946, bottled-wine by wine-at-bottling = -822, Bottled-wine by juice = -910.

For predictive patterns of bottled-wine constrained by patterns of Juice and Wine-at-bottling, respectively, are shown in panels (a) and (b) of Fig 8. Both partial coupling geometries reveal 4-block patterns, and their energy distributions are shown in panel (c) of Fig 5 (the red for Wine-at-bottling, blue for Juice). These two energy distributions are very close to the distribution under *H*_2*R*_ in the bottled-wine phase. Further their energies are marked upon the energy trajectory of the 2 × 2 checkerboard switching perturbation process, as shown in panel (c) of Fig 8. These evidences together confirm 4-block patterns with relative uniformity within each block. A similar predictive pattern from juice to wine-at-bottling is also concluded via its partial coupling geometry. Its corresponding energy distribution is seen being completely overlapping with that of *H*_3 × 2_. This linkages from juice to Wine-at-bottling, and then to bottled-wine are logical and natural within winemaking. As such, we demonstrate feasibility for Data Mechanics as a new computing paradigm and the integrative pattern inference as a pertinent testing approach for structural hypotheses.

The two large scale systemic factors of vintage and of biochemical measurements, and their interactions are confirmed through our integrative pattern inferences via partial coupling geometry computations. Hence we conclude that characteristics of grape, Juice and wines-at-bottling are expected to show up within bottled-wines. Since they are all holistic in nature, the next issue facing viticulturists and winemaker is: How to make good uses of such persistent patterns in winemakers? This issue in fact would force researchers to go away from typical “tuning one factor at a time” experimental design protocol. Thus any successful resolution to this issue is not only new, but also potentially fruitful and influential in many fields.

## Discussion

As an evolving system is expected to be embedded with unknown and complicate configurations of interacting dependence patterns, different computing paradigms and inferential techniques are needed to free researchers from constraints of independence. Our Data Mechanics is demonstrated being capable of successively extracting pattern information from a series of bipartite networks, and summarizing computed results into a series of coupling geometries. Then the integrative pattern inference, which is built upon network bootstrapping ensembles and their energy distributions based on multiscale block patterns contained in coupling geometries, is shown to allow scientists to address issues, such as: What is the geometry of each phase? How do serial phases evolve in terms of geometric patterns from causal and predictive perspectives? Discoveries are likely beyond winemaking.

In contrast, these issues are unlikely solvable by classic statistical methodologies, such as partial least square (PLS) and its variant regression techniques [6, 23]. The essential problem underlying these techniques is that they all rely on independence, normal distribution and linearity assumptions. However these assumptions hardly hold in any real world system. In other words, the primarily focus on linearity, such as based on variance-covariance matrices, likely is over-simplified, even unnaturally twisted.

As a further remark on significant merits of our data-driven inferential approach: our partial coupling geometry apparently resolves the issue of accommodating the high dimensional features of bipartite networks of covariates and their responses. Our energy distribution concept seems to summarize empirical significance from data. Hence our partial coupling geometry is more than an alternative to the popular and widely-used PLS or factor analyses. This new technique brings out multiscale pattern-to-pattern associations, i.e. the interacting dependence structures in a purely nonparametric fashion. We expect such nonparametric results being more reliable.

Finally, we remark why we focus on binary setting, instead of weighted one here. On one hand, in a binary setting, the data-driven computations are simpler and computed results are easier to visualize. On the other hand, in a weighted setting, both advantages are lessened. The primary barrier is caused by the current under-development in network bootstrapping on weighted bipartite networks. Through many computer experiments and the concept of a matrix as a coupling metric measure, it has become clear that simulating weighted matrix by subject to sequences of row and column sums like in a binary setting, as proposed in [24, 25] and references therein, is not correct. New and different algorithmic developments are needed for weighted setting.

## Supporting Information

S1 File Feature histograms and their thresholds Information for binary coding. In all four winemaking phases, a histogram is built for each feature and marked with one or two thresholds used for binary coding.
(PDF)

S2 File S2_File.xlsx, DOI: 10.6084/m9.figshare.3502373. Four binary data sets are contained in the File S2, an Excel file, for the four phases of winemaking: Harvest field, Juice, Wine at bottling (Prewine) and bottled Wine.
(XLSX)
