# Supplementary material for: Integrative Inferences on Pattern Geometries of Grapes Grown under Water Stress and Their Resulting Wines
Source: PLoS One. 2016 Aug 10;11(8):e0160621. doi: 10.1371/journal.pone.0160621 (PMC4980011; doi:10.1371/journal.pone.0160621)
Supplement: S1 File — In all four winemaking phases, a histogram is built for each feature and marked with one or two thresholds used for binary coding. (PDF) [file pone.0160621.s001.pdf]

S1 File : Feature histograms and their thresholds Information for binary coding

Supporting Information for "Integrative Inferences on Pattern Geometries of Grapes Grown under Water Stress and Their Resulting Wines"

Hsieh Fushing[1,*], Chih-Hsin Hsueh[1], Constantin Heitkamp[2] and Mark A. Matthews[2]

[1] Department of Statistics, University of California at Davis, CA, U.S.A

[2] Department of Viticulture and Enology, University of California at Davis, CA, U.S.A

* E-mail: fhsieh@ucdavis.edu

In this Supporting Information, we report all involved feature histograms and their thresholds Information for binary coding. In all four winemaking phases, a histogram is built for each feature and marked with one or two thresholds used for binary coding. Thresholds are chosen to be located within the valley when a feature-specific histogram is bimodal. When the bimodal distribution pattern is not obvious, thresholds are chosen to be located near the median of a histogram. The four sets of histograms superimposed with thresholds on the four phases are provided in following four figures below. When two thresholds are present on a histogram, these two thresholds give rather similar computational results. That is to say, our coupling geometry appears robust against small perturbations of thresholds.

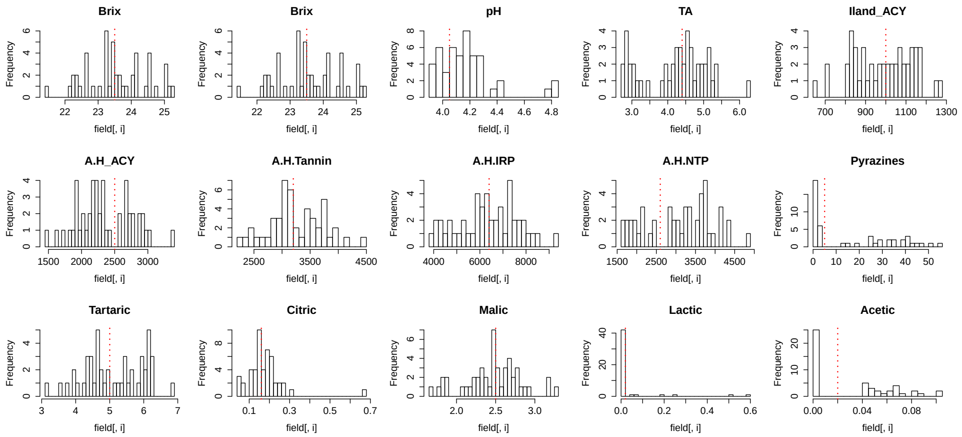

Figure A: Grape's histogram: Fifteen histograms with thresholds marked for binary coding grape composition data.

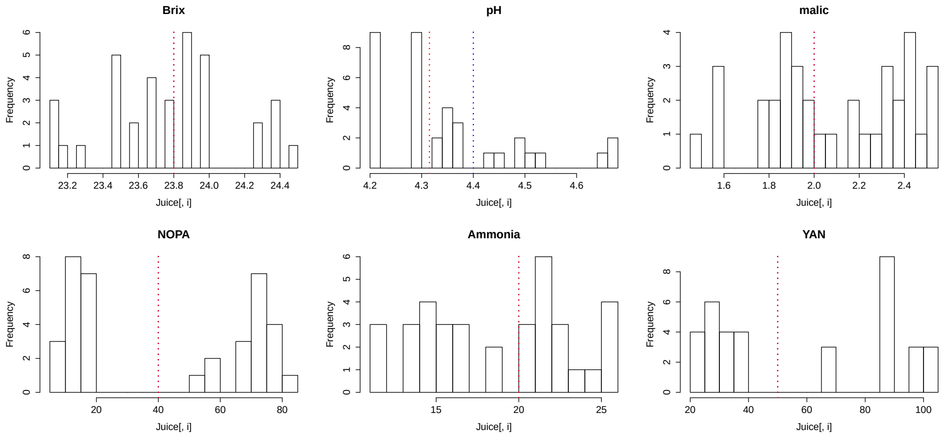

Figure B: Juice's Histogram: Six histograms with thresholds marked for binary coding juice data.

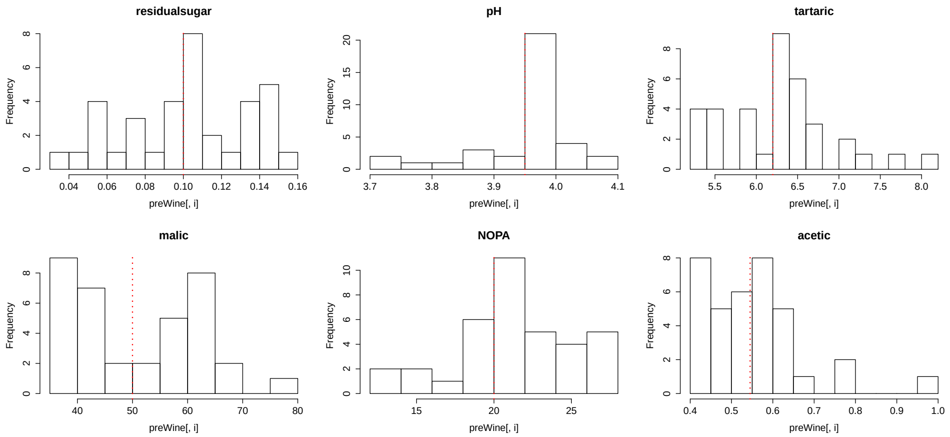

Figure C: Prewine's histograms: Six histograms with thresholds marked for binary coding of wine-at-bottling data.

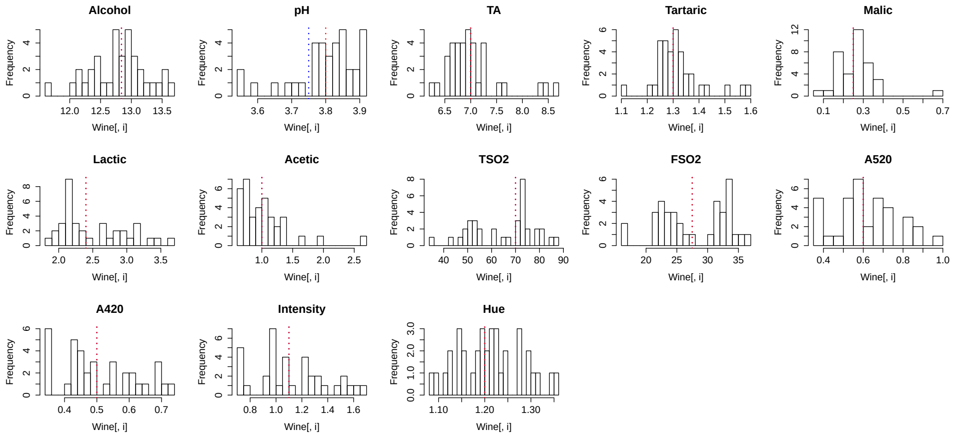

Figure D: Bottled wine's histogram:Thirteen histograms with thresholds marked for binary coding for bottled wine data.
